# Associations of Geriatric Nutritional Risk Index and Prognostic Nutritional Index With All‐Cause and Cardiovascular Mortality in DKD: NHANES 1999–2018

**DOI:** 10.1155/ije/5683927

**Published:** 2026-08-11

**Authors:** Yimei Li, Jimei Song, Liyan Liu, Wei Li, Qing Wang

**Affiliations:** ^1^ Department of Endocrinology, Kunshan Hospital of Chinese Medicine, Kunshan, China; ^2^ Department of Endocrinology, The First Affiliated Hospital of Guangzhou University of Chinese Medicine, Guangzhou, China, gzhmc.edu.cn; ^3^ Guangdong Clinical Research Academy of Chinese Medicine, Guangzhou, China; ^4^ Central Laboratory, Kunshan Hospital of Chinese Medicine, Kunshan, China; ^5^ Department of Orthopaedics, Kunshan Hospital of Chinese Medicine, Kunshan, China

**Keywords:** diabetic kidney disease, geriatric nutritional risk index, prognostic nutritional index

## Abstract

**Background:**

Malnutrition and immune status significantly influence the prognosis of diabetic kidney disease (DKD). However, the correlations between two novel nutritional–immune indices—geriatric nutritional risk index (GNRI) and prognostic nutritional index (PNI)—and the risk of all‐cause mortality (ACM) and cardiovascular mortality (CVM) in patients with DKD remain unclear. Our investigation was, therefore, designed to explore these associations.

**Methods:**

This study analyzed data from 2038 DKD individuals in the National Health and Nutrition Examination Survey (NHANES) (1999–2018). Multivariate weighted Cox regression, Kaplan–Meier survival curves (K–M curves), subgroup analysis, and interaction analysis were employed to clarify the associations of GNRI and PNI with ACM and CVM. We also applied restricted cubic splines (RCS) and threshold analysis to evaluate potential nonlinear correlations and inflection points. Subsequently, time‐dependent receiver operating characteristic (ROC) curves were utilized to explore and compare the prognostic performance of GNRI and PNI for survival outcomes over time.

**Results:**

Throughout the median follow‐up period of 68 months, 1006 (49.36%) of 2038 patients with DKD died, including 374 (37.18%) cardiovascular disease (CVD) deaths. Multivariate weighted Cox regression analysis and K–M curves illustrated that high levels of GNRI and PNI are independent protective factors against ACM and CVM in DKD patients. RCS analysis and threshold analysis revealed a nonlinear correlation of PNI with ACM and CVM, and the inflection points were identified at 51.24 and 49.63, respectively. What’s more, ROC curves demonstrated that GNRI and PNI exhibit significant predictive potential in both the short and long terms, with similar predictive performance.

**Conclusions:**

As novel nutritional–immune markers, GNRI and PNI could independently forecast the ACM and CVM in DKD patients, aiding in risk stratification and timely intervention for this disease.

## 1. Introduction

Diabetic kidney disease (DKD) is a prevalent and severe complication caused by long‐term diabetes mellitus (DM), influencing almost 20%–50% of T2DM patients [1]. The mortality risk of individuals with DM is approximately twice as high as that of nondiabetic individuals [2]. Research demonstrates that, in addition to glycemic control, the presence of DKD is a main determinant of excess risk of death in DM patients [3, 4], significantly increasing the all‐cause mortality (ACM) and cardiovascular mortality (CVM) rates in T2DM individuals [5]. Even within the normal range, higher albuminuria levels and lower estimated glomerular filtration rates (eGFR) indicate an elevated risk of mortality [6]. Therefore, exploring the prognostic factors in individuals with DKD is vital for assessing mortality risk and reducing DM‐related mortality.

Malnutrition is a considerable and critical concern in T2DM individuals, particularly those with DKD [7, 8]. Functioning as comprehensive assessments of nutritional status, immune function, and chronic inflammation, GNRI and PNI are composite biomarkers calculated from serum albumin, body weight, and lymphocyte counts [9, 10]. As such, the GNRI and PNI have emerged as simple and effective tools for evaluating nutritional status across numerous pathological conditions, offering more streamlined approaches compared to several other nutritional screening measures [11, 12]. Furthermore, researchers have suggested that GNRI and PNI correlate with the prognosis of several disorders, including T2DM and chronic kidney disease (CKD) [13–15]. A longitudinal study of 1100 T2DM patients illustrated that a decreased GNRI score is an important predictor for DKD progression [13]. Other studies have revealed that elevated PNI levels correlate with reduced ACM and CVM in diabetic or prediabetic cardiovascular disease (CVD) patients [14]. Additionally, PNI has been found to correlate with the occurrence of DKD and to be an independent indicator of ACM in T2DM patients [16]. While these findings suggest GNRI and PNI hold important prognostic value for DKD patients, current evidence remains limited by typically small sample sizes and insufficient data establishing definitive associations between these indices and ACM or CVM in DKD cohorts.

Therefore, this investigation sought to elucidate the relationships of GNRI and PNI with ACM and CVM among a nationally representative sample of individuals with DKD.

## 2. Materials and Methods

### 2.1. Data Source

**FIGURE 1:**
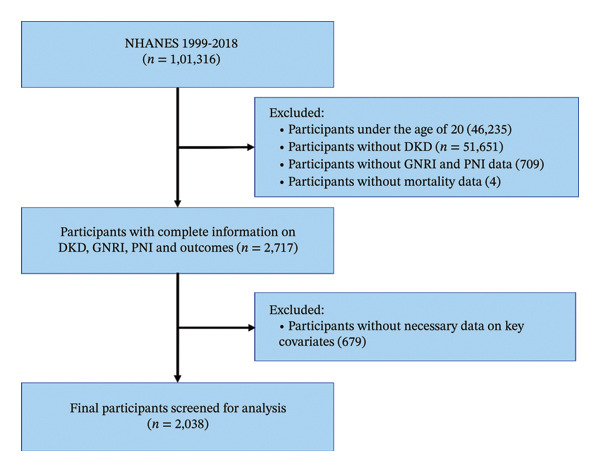
Flowchart. DKD, diabetic kidney disease; GNRI, geriatric nutritional risk index; PNI, prognostic nutritional index.

As a large‐scale, nationally representative survey, the National Health and Nutrition Examination Survey (NHANES) provides publicly available demographic, dietary, physical examination, laboratory, and questionnaire data [17]. Herein, we chose ten consecutive cycles from the NHANES database (1999–2000, 2001–2002, 2003–2004, 2005–2006, 2007–2008, 2009–2010, 2011–2012, 2013–2014, 2015–2016, and 2017–2018) as the basis for our analysis, which comprised 10,1316 participants. Participants younger than 20 years old and those without DKD were excluded from this study. Additionally, participants with incomplete data on the GNRI, PNI, ACM, CVM, and other necessary information (including complete blood count, biochemical tests, etc.) were also excluded. Ultimately, this investigation comprised 2,038 subjects (Figure 1).

### 2.2. Exposure and Outcome Variables

DM was defined as either meeting the American Diabetes Association criteria or having a self‐reported diagnosis or current use of hypoglycemic drugs [18]. DKD was identified per the KDIGO 2021 Guidelines [19]. Consistent with prior research, GNRI was measured as: GNRI = 1.489∗serum albumin + 41.7∗(actual body weight/ideal body weight), where ideal body weight = height − 100 − ((height − 150)/a), with *a* = 4 for males and 2.5 for females [20, 21].A GNRI cutoff of 98 classified participants as malnourished (GNRI < 98, reference) or well‐nourished (GNRI ≥ 98) [22]. PNI was calculated as follows: PNI = serum albumin + 5∗total lymphocyte. Besides, it was analyzed in quartiles, using the first quartile (Q1) as the reference group for comparison.

The study outcome variables included ACM and CVM. Mortality outcomes were ascertained from the NDI related to NHANES public‐use death files (1999–2018). Cardiovascular deaths were determined per the ICD‐10 codes.

### 2.3. Covariates

Covariates were determined according to the subsequent criteria: (1) demographic characteristics; (2) factors affecting exposure or outcome variables in line with previous studies; and (3) other potential variables accumulated in clinical experience. Eventually, a range of covariables were included: age, sex, ethnicity, education, alcohol intake, marital status, tobacco consumption, waist circumference (WC), body mass index (BMI), hypertension, dyslipidemia, blood urea nitrogen (BUN), serum creatinine (SCr), eGFR, aspartate aminotransferase (AST), alanine aminotransferase (ALT), glucose, HbA1c, hemoglobin, and total cholesterol (TC) level.

Participants were classified as never, former, or current smokers, with former and current smoking defined by a history of > 100 cigarettes. Alcohol intake was defined as ≥ 12 drinks in the year prior to the survey. BMI categories consisted of normal weight (< 25.0 kg/m^2^), overweight (25.0–30.0 kg/m^2^), and obese (≥ 30.0 kg/m^2^). eGFR was calculated using the CKD‐EPI equation. Hypertension was identified as diastolic BP ≥ 80 mmHg, systolic BP ≥ 130 mmHg, antihypertensive use, or self‐reported diagnosis [23]. Dyslipidemia was defined by abnormalities in lipid profiles (TC ≥ 5.18, TG ≥ 1.7, LDL ≥ 3.37, or sex‐specific low HDL) or self‐reported lipid‐lowering drug use [24]. Full details for these variables are publicly accessible in the NHANES database.

### 2.4. Statistical Analyses

In this research, R (v4.1.1) was utilized to conduct statistical analyses. Conforming to the recommendation of the NHANES protocol, the appropriate weighting methodology was applied to ensure nationally representative results [25]. We presented categorical data as proportions (95% CI) and continuous data as weighted means along with their standard errors, respectively, and compared them by weighted chi‐square and linear regression tests.

Survey‐weighted Cox proportional hazards models were utilized to assess correlations of GNRI and PNI with ACM and CVM risk, and we used three models to control for confounders. K–M curves were generated to compare survival probabilities across different GNRI and PNI categories. Subsequently, we employed subgroup and interaction analyses to assess consistency and robustness of the observed associations. To evaluate potential nonlinear relationships and inflection points, we performed restricted cubic spline (RCS) and threshold analysis, with the reference set at 98 for GNRI (the clinical cutoff for well‐nourished status) and at the median for PNI. Finally, the predictive accuracy of GNRI and PNI for mortality outcomes was evaluated and compared using time‐dependent receiver operating characteristic (ROC) curves. Additionally, given that serum albumin is a shared component of both GNRI and PNI, we examined its association with mortality risk using the same three Cox proportional hazards models as for GNRI and PNI. A two‐sided *p* value of less than 0.05 was considered statistically significant.

## 3. Results

### 3.1. Characteristics of Participants

As presented in Table 1, the study included 2038 NHANES participants, of whom 52.65% were male. Among them, the mean GNRI was 102.87 ± 0.15, and the mean PNI was 51.40 ± 0.19.

**TABLE 1:** Baseline characteristics.

Variables	Total (*n* = 2038)	ACM			CVM		
		No (*n* = 1032)	Yes (*n* = 1006)	*p*	No (*n* = 1664)	Yes (*n* = 374)	*p*
Age (years)	68.00 (60.00, 76.00)	65.00 (57.00, 72.00)	73.00 (64.00, 80.00)	**<** **0.001**	67.00 (60.00, 75.00)	72.00 (64.00, 80.00)	**<** **0.001**
Gender				0.870			0.208
Male	1073.00 (50.75)	514.00 (51.02)	559.00 (50.46)		858.00 (49.84)	215.00 (54.71)	
Female	965.00 (49.25)	518.00 (48.98)	447.00 (49.54)		806.00 (50.16)	159.00 (45.29)	
Ethnicity				**<** **0.001**			0.124
Mexican American	396.00 (7.21)	226.00 (8.90)	170.00 (5.42)		340.00 (7.69)	56.00 (5.16)	
Other Hispanic	162.00 (4.95)	109.00 (6.02)	53.00 (3.81)		143.00 (5.10)	19.00 (4.31)	
Non‐Hispanic White	803.00 (64.67)	315.00 (58.44)	488.00 (71.28)		618.00 (63.21)	185.00 (71.01)	
Non‐Hispanic Black	557.00 (16.22)	301.00 (18.38)	256.00 (13.93)		453.00 (16.45)	104.00 (15.22)	
Other race	120.00 (6.94)	81.00 (8.25)	39.00 (5.55)		110.00 (7.55)	10.00 (4.30)	
Educational level				**<** **0.001**			**0.006**
Less than high school	895.00 (33.60)	406.00 (27.88)	489.00 (39.68)		256.00 (14.69)	76.00 (19.70)	
High school or equivalent	469.00 (25.66)	244.00 (25.87)	225.00 (25.43)		495.00 (27.28)	116.00 (26.78)	
Above high school	674.00 (40.74)	382.00 (46.25)	292.00 (34.89)		913.00 (58.03)	182.00 (53.51)	
BMI (kg/m^2^)				**<** **0.001**			0.163
< 25	332.00 (15.63)	134.00 (11.81)	198.00 (19.68)		256.00 (14.69)	76.00 (19.70)	
25–30	611.00 (27.19)	280.00 (25.24)	331.00 (29.25)		495.00 (27.28)	116.00 (26.78)	
≥ 30	1095.00 (57.19)	618.00 (62.94)	477.00 (51.08)		913.00 (58.03)	182.00 (53.51)	
Alcohol intake				0.209			0.307
Yes	1200.00 (59.33)	609.00 (61.18)	591.00 (57.38)		983.00 (59.97)	217.00 (56.57)	
Smoking status				**0.048**			0.418
Never smoker	918.00 (44.41)	495.00 (47.96)	423.00 (40.65)		753.00 (44.99)	165.00 (41.93)	
Former smoker	817.00 (40.82)	377.00 (37.53)	440.00 (44.31)		666.00 (40.90)	151.00 (40.47)	
Current smoker	303.00 (14.77)	160.00 (14.51)	143.00 (15.04)		245.00 (14.12)	58.00 (17.60)	
Marital status				**<** **0.001**			**0.035**
Married or living with a partner	1072.00 (55.66)	576.00 (60.08)	496.00 (50.96)		894.00 (57.12)	178.00 (49.32)	
Others	966.00 (44.34)	456.00 (39.92)	510.00 (49.04)		770.00 (42.88)	196.00 (50.68)	
Hypertension				**0.008**			0.821
Yes	1736.00 (84.59)	867.00 (81.70)	869.00 (87.65)		1417.00 (84.46)	319.00 (85.15)	
Hyperlipidemia				0.686			0.690
Yes	1820.00 (89.90)	925.00 (89.56)	895.00 (90.26)		1485.00 (89.73)	335.00 (90.64)	
Serum albumin (g/dL)	4.12 (0.01)	4.16 (0.01)	4.07 (0.01)	**<** **0.001**	4.13 (0.01)	4.05 (0.02)	**<** **0.001**
ALT (U/L)	21.00 (16.00, 28.00)	22.00 (17.00, 31.00)	19.00 (16.00, 25.00)	**<** **0.001**	21.00 (16.00, 29.00)	19.00 (16.00, 25.00)	**0.010**
AST (U/L)	23.00 (19.00, 29.00)	23.00 (20.00, 29.00)	23.00 (19.00, 28.00)	0.223	23.00 (19.00, 29.00)	22.00 (19.00, 28.00)	0.177
BUN (mmol/L)	6.43 (5.00, 8.93)	6.07 (4.64, 7.85)	7.14 (5.36, 9.64)	**<** **0.001**	6.43 (4.64, 8.57)	7.10 (5.36, 9.28)	**0.005**
SCr (μmol/L)	97.24 (75.14, 123.76)	94.59 (73.37, 117.57)	99.01 (79.56, 129.06)	**<** **0.001**	97.24 (75.14, 121.99)	99.01 (79.56, 124.64)	0.303
eGFR (mL/min/1.73m2)	63.48 (48.01, 90.80)	67.78 (51.12, 96.05)	59.05 (44.43, 83.66)	**<** **0.001**	63.88 (48.41, 92.25)	62.29 (47.06, 84.36)	0.262
Glucose (mmol/L)	7.44 (5.94, 9.99)	7.44 (6.05, 10.21)	7.38 (5.77, 9.83)	0.218	7.44 (6.05, 9.99)	7.38 (5.61, 10.21)	0.251
HbA1c (%)	6.90 (6.20, 8.00)	7.00 (6.30, 8.10)	6.80 (6.10, 7.90)	**0.005**	6.90 (6.20, 8.00)	6.80 (6.20, 8.10)	0.638
Hemoglobin (g/L)	13.75 (0.06)	13.86 (0.08)	13.64 (0.07)	**0.031**	13.75 (0.06)	13.76 (0.10)	0.976
TC (mmol/L)	4.84 (0.03)	4.82 (0.05)	4.86 (0.05)	0.638	4.84 (0.04)	4.83 (0.07)	0.869
WC (cm)	109.76 (0.53)	111.05 (0.63)	108.40 (0.75)	**0.004**	110.03 (0.52)	108.59 (1.27)	0.257
GNRI	102.87 (0.15)	103.61 (0.19)	102.09 (0.20)	**<** **0.001**	103.12 (0.16)	101.77 (0.32)	**<** **0.001**
PNI	51.40 (0.19)	51.93 (0.17)	50.84 (0.32)	**0.001**	51.55 (0.20)	50.77 (0.42)	0.078

*Note:* CVM, cardiovascular mortality; AST, aspartate aminotransferase; ALT, alanine aminotransferase; SCr, serum creatinine; HbA1c, hemoglobin A1c; TC, total cholesterol level. The bold values indicate the statistical results with *p* < 0.05.

Abbreviations: ACM, all‐cause mortality; BMI, body mass index; BUN, blood urea nitrogen; eGFR, estimated glomerular filtration rate; WC, waist circumference.

Compared to the no ACM group, the ACM group comprised older individuals with higher BUN and SCr levels, and a greater proportion of smokers and hypertensive patients. The distribution of ethnicity also differed significantly between groups. Specifically, non‐Hispanic White individuals constituted a larger proportion within the ACM group. Moreover, people in the ACM group had lower levels of serum albumin, eGFR, and hemoglobin, lower educational attainment, and rates of marriage or cohabitation (all *p* < 0.05). Similarly, compared to the no CVM group, older average age, higher BUN level, as well as lower serum albumin level, rates of marriage or cohabitation, and educational attainment were more common among the CVM group (all *p* < 0.05). Interestingly, compared to the no ACM and no CVM groups, individuals in the ACM and the CVM groups tended to have reduced GNRI and PNI values, indicating a potentially reduced nutritional status in these groups.

### 3.2. Associations of GNRI and PNI With ACM Among American Adults With DKD

**FIGURE 2:**
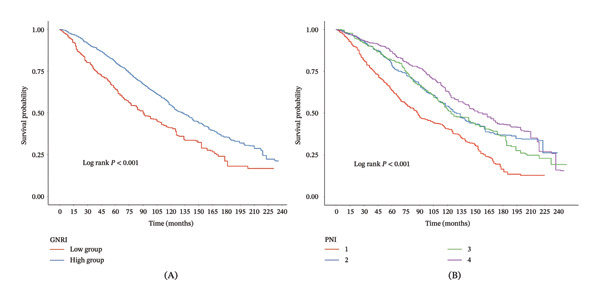
Kaplan–Meier survival curves. (A) ACM vs. GNRI; (B) ACM vs. PNI. ACM, all‐cause mortality; GNRI, geriatric nutritional risk index; PNI, prognostic nutritional index.

During a median 68‐month follow‐up (IQR: 51–126), 1006 DKD patients (49.36%) died, including 374 (37.18%) CVM cases. The weighted Cox regression analysis (Table 2) showed that higher GNRI and PNI levels, whether considered as continuous or categorical variables, were significantly linked to lower ACM risk. In the crude model, GNRI was inversely associated with ACM (continuous variable: HR = 0.95, 95% CI: 0.94–0.97, *p* < 0.001; categorical variable: high‐GNRI group, 0.61, 0.49–0.75, *p* < 0.001), with significance maintained after full adjustment. Similarly, in the unadjusted model, each 1‐point PNI increase reduced ACM risk by 4% (0.96, 0.94–0.98, *p* < 0.001), and higher categorical PNI level were a strongly protective factor for ACM (top quartile: 0.45, 0.37–0.56, *p* < 0.001). The association also remained robust after considering confounding factors. K–M curves showed similar tendencies, with higher GNRI and PNI levels being tied to a reduced risk of ACM (Figure 2). Notably, serum albumin was also significantly associated with ACM across all three adjustment models (0.52, 0.42–0.63, *p* < 0.001; Table 2).

**TABLE 2:** Associations between GNRI, PNI and mortality in DKD.

Variables	Model 1		Model 2		Model 3	
	HR (95% CI)	*p*	HR (95% CI)	*p*	HR (95% CI)	*p*
*ACM*						
GNRI	0.95 (0.94–0.97)	**<** **0.001**	0.94 (0.92–0.95)	**<** **0.001**	0.95 (0.93–0.96)	**<** **0.001**
GNRI categories						
Low‐GNRI group	1.00 (Reference)		1.00 (Reference)		1.00 (Reference)	
High‐GNRI group	0.61 (0.49–0.75)	**<** **0.001**	0.49 (0.40–0.61)	**<** **0.001**	0.58 (0.45–0.73)	**<** **0.001**
PNI	0.96 (0.94–0.98)	**<** **0.001**	0.96 (0.94–0.99)	**0.001**	0.97 (0.95–0.99)	**0.030**
PNI categories						
Q1	1.00 (Reference)		1.00 (Reference)		1.00 (Reference)	
Q2	0.57 (0.45–0.72)	**<** **0.001**	0.59 (0.46–0.75)	**<** **0.001**	0.62 (0.49–0.78)	**<** **0.001**
Q3	0.60 (0.49–0.72)	**<** **0.001**	0.64 (0.53–0.77)	**<** **0.001**	0.66 (0.54–0.79)	**<** **0.001**
Q4	0.45 (0.37–0.56)	**<** **0.001**	0.51 (0.42–0.63)	**<** **0.001**	0.56 (0.46–0.70)	**<** **0.001**
Serum albumin	0.52 (0.42–0.63)	**<** **0.001**	0.41 (0.33–0.51)	< 0.001	0.48 (0.39–0.60)	**<** **0.001**

*CVM*						
GNRI	0.94 (0.93–0.96)	**<** **0.001**	0.93 (0.91–0.95)	**<** **0.001**	0.93 (0.91–0.96)	**<** **0.001**
GNRI categories						
Low‐GNRI group	1.00 (Reference)		1.00 (Reference)		1.00 (Reference)	
High‐GNRI group	0.49 (0.36–0.67)	**<** **0.001**	0.40 (0.29–0.56)	**<** **0.001**	0.43 (0.30–0.60)	**<** **0.001**
PNI	0.96 (0.93–0.99)	**0.004**	0.96 (0.93–0.99)	**0.024**	0.96 (0.93–0.99)	**0.037**
PNI categories						
Q1	1.00 (Reference)		1.00 (Reference)		1.00 (Reference)	
Q2	0.69 (0.48–0.99)	**0.045**	0.71 (0.50–1.02)	0.064	0.72 (0.50–1.05)	0.092
Q3	0.54 (0.39–0.76)	**<** **0.001**	0.59 (0.43–0.83)	**0.002**	0.58 (0.41–0.83)	**0.002**
Q4	0.51 (0.37–0.72)	**<** **0.001**	0.59 (0.43–0.83)	**0.002**	0.58 (0.40–0.84)	**0.004**
Serum albumin	0.44 (0.34–0.58)	**<** **0.001**	0.35 (0.26–0.48)	**<** **0.001**	0.37 (0.26–0.52)	**<** **0.001**

*Note:* Model 1 was unadjusted; Model 2 adjusted for gender, age, race, BMI; Model 3 further adjusted for educational level, alcohol intake, marital status, smoking status, WC, history of hypertension and dyslipidemia, as well as levels of ALT, AST, TC, eGFR, BUN, SCr, glucose, hemoglobin, and HbA1c. The bold values indicate the statistical results with *p* < 0.05.

**FIGURE 3:**
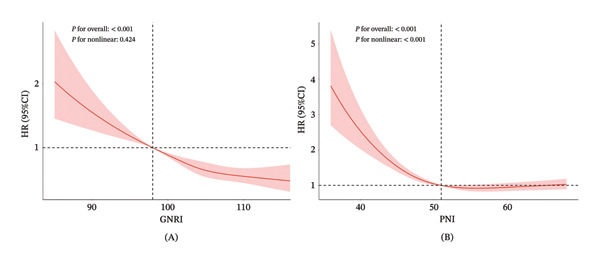
Restricted cubic splines. (A) GNRI vs. ACM; (B) PNI vs. ACM. ACM, all‐cause mortality; GNRI, geriatric nutritional risk index; PNI, prognostic nutritional index.

RCS analysis, commonly used for data exploration to illustrate potential nonlinear correlations between exposure and outcome variables, was conducted in this study. It was found that in patients with DKD, PNI exhibited a negative and L‐shaped nonlinear correlation with ACM (nonlinearity of *p* < 0.001). The analysis did not reveal significant evidence of a nonlinear relationship between GNRI and ACM (*p* = 0.424; Figure 3). Subsequently, subgroup analyses were performed across key demographic, clinical, and laboratory variables, which demonstrated reliable relationships of GNRI and PNI with ACM. In the interaction analysis, no significant interactions between the above factors and GNRI were discovered for ACM (all *p* interaction > 0.05). In contrast, statistically significant interactions of gender and BMI with PNI on ACM were identified (*p* interaction > 0.05). Details of the subgroup and interaction analyses are provided in the Supporting Figures S1 and S2.

### 3.3. Associations of GNRI and PNI With CVM Among American Adults With DKD

**FIGURE 4:**
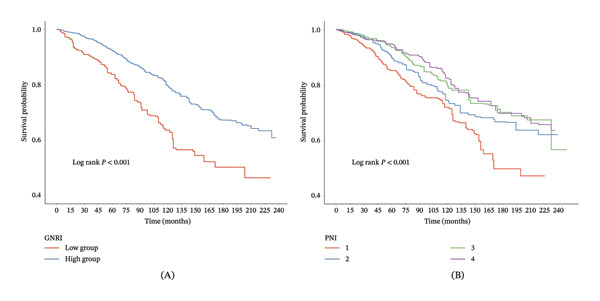
Kaplan–Meier survival curves. (A) CVM vs. GNRI; (B) CVM vs. PNI. CVM, cardiovascular mortality; GNRI, geriatric nutritional risk index; PNI, prognostic nutritional index.

Likewise, weighted Cox regression analysis demonstrated that elevated GNRI and PNI levels, whether assessed as continuous or categorical variables, were significantly related to decreased risk of CVM (Table 2). Specifically, in the crude model, each 1‐point increment in GNRI as a continuous variable corresponded to a 6% decrease in CVM risk (0.94, 0.93–0.96, *p* < 0.001). Upon stratifying GNRI into quartiles, the high GNRI group exhibited a 51% lower CVM risk than the low GNRI group (0.49, 0.36–0.67, *p* < 0.001), with this link persisting in Model 3 after adjustment for confounders. Parallel findings were observed for the correlation between PNI and CVM in DKD individuals. High PNI levels were indicative of significantly lower CVM risks (PNI as a continuous variable: 0.96, 0.93–0.99, *p* = 0.004; PNI as a categorical variable: the highest quartile, 0.51, 0.37–0.72, *p* < 0.001). K–M curves also suggested that higher GNRI and PNI levels were associated with lower CVM risks (Figure 4). In addition, serum albumin showed an inverse association with CVM in all three models (0.44, 0.34–0.58, *p* < 0.001; Table 2).

**FIGURE 5:**
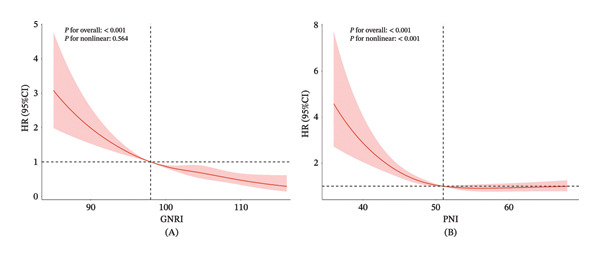
Restricted cubic splines. (A) GNRI vs. CVM; (B) PNI vs. CVM. CVM, cardiovascular mortality; GNRI, geriatric nutritional risk index; PNI, prognostic nutritional index.

Furthermore, RCS analysis unveiled a nonlinear association between PNI and CVM (nonlinearity of *p* < 0.001). However, there remained insufficient evidence to support a nonlinear correlation between GNRI and CVM (*p* = 0.564) (Figure 5). In the subgroup analyses stratified by a series of potential confounding factors, the correlations of GNRI and PNI with CVM remained consistent. Interaction analysis revealed notable interactions of gender and hyperlipidemia with GNRI on CVM (*p* interaction > 0.05) whereas for PNI, significant interactions on CVM were also observed with gender, educational level, alcohol status, and hyperlipidemia (*p* interaction > 0.05). Details of the subgroup and interaction analyses are provided in the Supporting Figures S3 and S4.

### 3.4. Threshold Analysis

Based on the RCS findings, to characterize the potential nonlinear association between PNI and mortality and to determine the inflection point, a threshold effect analysis was employed (Table 3). The results revealed that PNI exhibited inverse relationships with both ACM and CVM, with inflection points at 51.24 and 49.63, respectively. Under these cut‐points, the increase in PNI corresponded to a consistent decline in mortality risk (ACM: 0.92, 0.89–0.94, *p* < 0.001; CVM: 0.89, 0.85–0.94, *p* < 0.001). Conversely, once PNI exceeded these benchmarks, the protective effect plateaued and was no longer significant (*p* > 0.05).

**TABLE 3:** Threshold analysis of PNI and mortality.

Outcome	Effect	*p*
ACM		
Inflection point	51.24	
< 51.24	0.92 (0.89–0.94)	**<** **0.001**
≥ 51.24	1.01 (1.00–1.02)	0.068
*p*		**<** **0.001**
CVM		
Inflection point	49.63	
< 49.625	0.89 (0.85–0.94)	**<** **0.001**
≥ 49.625	1.00 (0.98–1.02)	0.866
*p*		**<** **0.001**

*Note:* The bold values indicate the statistical results with *p* < 0.05.

### 3.5. Prognostic Value of GNRI and PNI

**FIGURE 6:**
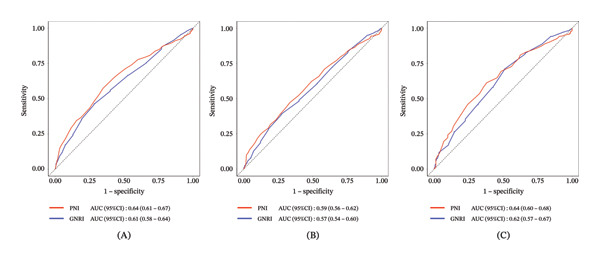
Assessment of GNRI and PNI predicting performance for mortality based on time‐dependent receiver operating characteristic curves. (A) 5 years; (B) 10 years; (C) 15 years. GNRI, geriatric nutritional risk index; PNI, prognostic nutritional index.

Employing time‐dependent ROC curves from the crude model, we compared the predictive performance of GNRI and PNI for ACM and CVM (Figure 6). The findings revealed that, in terms of ACM, the AUC for GNRI was 0.61 (0.58–0.64) at 5 years, 0.57 (0.54–0.60) at 10 years, and 0.62 (0.57–0.67) at 15 years. For PNI, the AUC values were 0.64 (0.61–0.67) at 5 years, 0.59 (0.56–0.62) at 10 years, and 0.64 (0.60–0.68) at 15 years. According to these results, both GNRI and PNI demonstrate significant predictive potential for ACM and CVM in both the short and long terms, with similar predictive abilities.

## 4. Discussion

So far as the literature indicates, no study has comprehensively explored the connections of GNRI and PNI with mortality among DKD patients. Our analyses illustrated that higher GNRI and PNI levels were significantly correlated with reduced ACM and CVM risks, with a nonlinear link observed between PNI and mortality. These associations remained consistent across all prespecified subgroups, including demographic, clinical, and laboratory factors. Furthermore, time‐dependent ROC analysis showed that both GNRI and PNI reliably predicted 5‐, 10‐, and 15‐year survival rates for ACM and CVM, with comparable prognostic performance.

An estimated 40% of individuals with diabetes will progress to DKD, a major microvascular complication of the disease. Moreover, the mortality risk of DKD remains high, making it one of the top ten causes of death worldwide [26]. According to previous research, DKD is a metabolic disorder associated with chronic inflammation, which has been reported to potentially accelerate malnutrition and immune dysfunction, thereby promoting inflammatory responses and creating a vicious cycle that drives disease progression and adverse outcomes [27]. Therefore, nutritional–immune indices that can accurately reflect the individual’s nutritional and immune status may act as effective prognostic indicators for forecasting ACM and CVM in DKD individuals.

Herein, the levels of GNRI and PNI were negatively related to ACM and CVM in DKD patients, indicating that elevated GNRI and PNI levels serve as protective factors. This underlines the important role of optimizing nutritional and immune status to improve DKD outcomes. A longitudinal study involving 1940 Korean patients with T2DM illustrated that a low GNRI score is an important predictor for CKD progression [13]. Another prospective survey linked lower GNRI to increased risks of renal events (5.15, 2.51–10.60) and ACM (2.30, 1.20–4.42) in Japanese patients with T2DM [28]. Similar findings have also been reported in studies on PNI. A retrospective cohort study from China showed that PNI is related to eGFR and glomerular damage, and serves as an independent indicator of DKD progression in T2DM individuals, outperforming anemia and hypoalbuminemia in predictive capacity [15]. Zhang et al., utilizing NHANES data, found that higher PNI values in T2DM patients are independently linked to a low risk of DKD (0.64, 0.459–0.892) and lower ACM (0.60, 0.37–0.97) [16]. Additionally, another NHANES study illustrated that high PNI is significantly related to decreased ACM and CVM risks in CVD individuals with DM or pre‐DM, specifically for CVD patients with DM [14]. The aforementioned studies collectively indicate the potential prognostic value of GNRI and PNI for DKD outcomes. For the first time, our study validates this hypothesis and demonstrates that GNRI and PNI possess comparable predictive abilities in forecasting ACM and CVM risks in DKD patients.

The GNRI and PNI are composite nutritional indicators that both incorporate serum albumin, a critical biomarker for assessing systemic nutritional status [29]. Recent studies have identified that reduced levels of serum albumin may serve as an independent risk indicator for the presence and progression of DKD [30]. Furthermore, serum albumin may counteract key DKD pathogenic drivers—oxidative stress and inflammation [31]—by functioning as an extracellular antioxidant and an anti‐inflammatory agent that modulates glutathione and TNF‐*α*/NF‐κB signaling [32, 33]. Given these biological functions, we also examined the association of serum albumin alone with mortality risk. As shown in Table 2, higher albumin levels were significantly associated with lower ACM and CVM. The numerically smaller hazard ratios for albumin may partly reflect the difference in units (per 1 g/dL for albumin vs. per one point for the indices). Furthermore, GNRI and PNI are composite measures that integrate albumin with body weight (GNRI) or lymphocyte count (PNI), thereby capturing a more comprehensive picture of a patient’s nutritional status. As such, they may serve as more practical standardized tools for risk stratification in DKD patients. Thus, rather than replacing albumin in clinical practice, GNRI and PNI complement it by offering a more holistic nutritional assessment. Body weight is another component of the GNRI. A Swedish population‐based case‐control study indicated that the coexistence of obesity and DM doubles the risk of developing kidney disease [34, 35]. Investigations have found that obesity can trigger a low‐grade inflammatory state and macrophage infiltration into the kidneys, inducing the release of proinflammatory factors and thereby exacerbating DM‐associated glomerulopathy [36]. As another key component of the PNI, lymphocytes can reflect the body’s immune function, with reduced lymphocyte counts indicating immunosuppression or dysfunction [37]. Research has shown a significant association between decreased serum lymphocyte counts and DKD. Given that chronic inflammation is a key driver of DKD progression, reduced lymphocyte counts may further aggravate the condition by impairing inflammation control and tissue repair [38]. Thus, considering the interplay among metabolic dysfunction, inflammation, and immune response in DKD, GNRI, and PNI—which integrate body weight, serum albumin, and lymphocyte levels—show potential as valuable prognostic tools.

In the current research, we discovered that PNI was nonlinearly related to both ACM and CVM, and the thresholds of 51.24 and 49.63 were identified, respectively. Below these values, the increase in PNI corresponded to a decline in the risk of death. A cross‐sectional investigation of 1,509 participants demonstrated that, in CVD patients with DM or pre‐DM, PNI was nonlinearly correlated with mortality, with a cut‐off of 46.5 determined [14], which was very similar to the threshold ascertained in our study. In contrast, RCS analysis did not reveal a significant nonlinear link between GNRI and either ACM or CVM (*p* > 0.05). This suggests that, given the current sample size and statistical model, the correlation between GNRI and death is more likely to be linear. The lack of a significant nonlinear correlation in the RCS analysis may be attributable to several factors. First, unmeasured confounding factors may have influenced the correlation between GNRI and mortality. Second, the actual correlation may be linear, or the nonlinear relationship may be too weak to be detected with the current sample size and statistical methods. Hence, further exploration is needed in future studies.

A key strength of this study is its large, diverse sample, enhancing both statistical power and robustness of our findings. Additionally, recruiting all participants from the NHANES database inherently minimizes selection bias, and the reliability of death outcomes was further strengthened by the extended follow‐up period. Furthermore, our research provides a novel contribution to the field by highlighting GNRI and PNI as valuable prognostic markers for ACM and CVM in individuals with DKD.

Nevertheless, several limitations of this study warrant mention. First, although multiple potential confounders were considered, unmeasured variables may still have influenced the results. Second, as participants were drawn exclusively from the United States, the generalizability of the results to other populations requires further investigation. Third, due to the cross‐sectional design with only baseline data, the predictive value of GNRI and PNI over time, along with any causal inferences, remains uncertain.

## 5. Conclusions

After analyzing 2038 individuals with DKD from NHANES (1999–2018), our investigation uncovered significant inverse correlations of GNRI and PNI with ACM and CVM. The findings provide robust evidence for incorporating GNRI and PNI as effective prognostic markers of mortality into routine clinical assessments for DKD patients and highlight the significant role of nutrition and immunity in improving DKD outcomes.

## Author Contributions

Yimei Li: conceptualization, data curation, and formal analysis. Jimei Song: formal analysis and writing–original draft. Liyan Liu: conceptualization, methodology, and writing–review and editing. Wei Li: conceptualization, supervision, and writing–review and editing. Qing Wang: methodology, supervision, and project administration.

## Funding

This study was supported by Suzhou Science and Technology Plan (Grant No. SYWD2025222).

## Disclosure

All authors have read and agreed to the published version of the manuscript.

## Ethics Statement

The studies involving human participants were reviewed and approved by the NCHS Research Ethics Review Board. The participants provided their written informed consent to participate in this study. The studies were conducted in accordance with the local legislation and institutional requirements.

## Conflicts of Interest

The authors declare no conflicts of interest.

## Supporting Information

Additional supporting information can be found online in the Supporting Information section.

## Supporting information


**Supporting Information 1** Supporting Figure S1: Subgroup and interaction analyses for the GNRI‐ACM association.

**Supporting Information 2** Supporting Figure S2: Subgroup and interaction analyses for the PNI‐ACM association.

**Supporting Information 3** Supporting Figure S3: Subgroup and interaction analyses for the GNRI‐CVM association.

**Supporting Information 4** Supporting Figure S4: Subgroup and interaction analyses for the PNI‐CVM association.

## Data Availability

The study data are available from the corresponding author on request and remain nonpublic to safeguard patients’ privacy rights.
