# Supplementary material for: Associations of Geriatric Nutritional Risk Index and Prognostic Nutritional Index With All‐Cause and Cardiovascular Mortality in DKD: NHANES 1999–2018
Source: Int J Endocrinol. 2026 Aug 11;2026:5683927. doi: 10.1155/ije/5683927 (PMC13462659; doi:10.1155/ije/5683927)
Supplement: Supplementary file 3 [file IJE-2026-5683927-s003.docx]

Supplementary Figure S1: Subgroup and interaction analyses for the GNRI-ACM association.
